# Emanuel Syndrome: A Case Report With Isolated Nuchal Translucency Thickening

**DOI:** 10.1155/crig/5541504

**Published:** 2025-10-08

**Authors:** Bondarenko Maya, Nikolenko Marharyta, Dutchak Anastasiia, Kupchak Iryna, Ivanova Irina, Arbuzova Svitlana

**Affiliations:** ^1^Department of Medical Biology and Medical Genetics, Ivano-Frankivsk National Medical University, 2 Galitska Str., Ivano-Frankivsk 76000, Ukraine; ^2^Eastern-Ukrainian Center for Medical Genetics and Prenatal Diagnosis, Mariupol & Kyiv, 23 Kopernika Str., Kyiv 02000, Ukraine; ^3^Faculty of Health and Life Sciences, University of Exeter, Northcote House, The Queen's Drive, Exeter, Devon EX4 4QJ, UK

**Keywords:** Emanuel syndrome, nuchal translucency, prenatal diagnosis

## Abstract

**Introduction:**

Emanuel syndrome is a rare chromosomal disorder characterized by severe developmental disability and variable clinical manifestations. Although congenital anomalies are relatively common, there is no pathognomonic prenatal pattern. In some cases, structural defects are absent or not detectable prenatally, making the potential role of soft ultrasound markers particularly relevant.

**Case Presentation:**

We report a case of Emanuel syndrome in which no structural malformations were identified prenatally. First-trimester ultrasound revealed an isolated increased nuchal translucency of 3.2 mm. Postnatally, the infant exhibited severe hypotonia, dysmorphic features, and profound developmental delay, but no gross structural defects were observed. Cytogenetic and FISH analyses confirmed an additional der(22)t(11; 22) chromosome inherited from the mother.

**Discussion:**

A review of the limited literature on first-trimester findings suggests that increased nuchal translucency has been observed in several cases of Emanuel syndrome, although the available data remain insufficient to assess predictive value. Nevertheless, the recurrence of this observation across independent reports indicates that NT enlargement may warrant attention as a potential prenatal marker.

**Conclusion:**

While current evidence is insufficient to draw definitive conclusions, this case highlights that isolated NT thickening may represent the only prenatal sign of Emanuel syndrome. Evaluation in larger cohorts and future prospective studies will be essential to determine the sensitivity and specificity of this marker for early diagnosis of the syndrome and the timely identification of balanced translocation carriers.

## 1. Introduction

Emanuel syndrome (ES; OMIM #609029) is a rare genetic disorder resulting predominantly from a 3:1 meiotic malsegregation of a parental balanced translocation t(11; 22)(q23; q11) [1, 2]. Over 99% of cases involve maternal carriership of this translocation, one of the most common recurrent non-Robertsonian translocations in humans [3–5]. ES is characterized by significant developmental delay, intellectual disability, facial dysmorphism, and variable congenital anomalies more often affecting the cardiovascular (∼50%), renal (∼25%), and skeletal systems (∼40%) [3–10]. Some patients exhibit mild congenital defects [3–7, 9, 10], further complicating timely diagnosis. The syndrome was diagnosed in the cohort of 63 patients within the first month of the child's life in 48% [4]; in the study of 43 individuals, ES features were well evident in approximately 30% of cases [3].

There is a prevailing belief that despite some common ES features, routine second-trimester prenatal screening lacks sufficient sensitivity, largely due to the variability of ultrasound findings often not evident prenatally. While facial features, such as malformed ears with pits and tags, cleft palate, micrognathia, and flat nasal bridge are typical, they are inconsistently present and may not be echographically visible [11–23]. However, the diagnostic potential of early screening in the first trimester remains underexplored.

We present a case of ES with subtle prenatal and postnatal manifestations. The distinctive prenatal feature was increased nuchal translucency (NT). In this report, we explore the informativeness of first-trimester screening for ES, with a focus on NT as a potential marker.

## 2. Case Presentation

A 32-year-old gravida 2, para 1 woman delivered a female newborn weighing 2990 g at 40 weeks via Caesarean section. The father's age at the time of delivery was 34 years. The parents, unrelated and without a history of congenital anomalies or mental retardation, had a healthy first child.

The pregnancy was complicated by first-trimester placental detachment and a 34-week maternal COVID-19 infection. First-trimester screening identified the patient as high-risk for trisomy 21, with NT measuring 3.2 mm (2.1 MoM), while biochemical markers (PAPP-A: 0.76 MoM, hCG: 1.56 MoM) were unremarkable. The mother declined invasive testing. Second-trimester ultrasound at 20 weeks revealed placenta previa, moderate oligohydramnios, and a hyperechoic focus in the left ventricle. No gross malformations were detected. Biochemical markers were also within normal limits: AFP—0.87 MoM, hCG—2.4 MoM, and Estriol—1.13 MoM. At the 30 weeks of ultrasound scan, signs of fetal development delay appeared.

**Figure 1:**
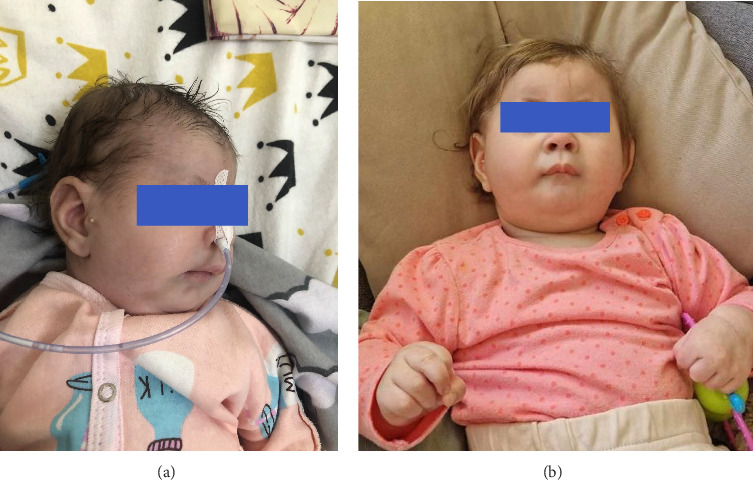
Facial features of the child (a) at birth; (b) at 11 months.

At birth, phenotypic signs included slight facial asymmetry, micrognathia, upward-slanting palpebral fissures, epicanthus and strabismus, short philtrum, wide bridge of nose, open nostrils, high-arched palate, enlarged low-set ears with a solitary right-sided preauricular tag, and a small first toe. The infant exhibited severe hypotonia, feeding difficulties, respiratory problems, and needed artificial ventilation (Figure 1(a)).

At 11 months (Figure 1(b)), the child demonstrated profound developmental delay and convulsive syndrome (periodic episodes of uncontrollable muscle contractions/seizures). She was unable to sit or crawl and could not control her head, had a microcephalic skull shape, and bilateral dislocation of the hip joints. The child reacted poorly to sounds, and hearing assessment revealed right-sided hearing loss. Brain MRI revealed only mild hypoplasia of the hippocampus and cerebellar vermis without any major anomalies. The girl was also diagnosed with moderate hypoplasia of the left kidney.

### 2.1. Cytogenetic and Molecular Analysis

Conventional G-banding chromosome analysis following a standard protocol (400–550-band resolution) and molecular cytogenetic analysis fluorescent in situ hybridization (FISH) were provided. Metaphase plates were analyzed using IKAROS V5.7 software (MetaSystems Hard & Software GmbH, Germany) and a light microscope (Axioscope, АХIO Imager M2, Carl Zeiss Jena GmbH). FISH analysis was performed on interphase nuclei according to the protocol recommended by the manufacturer of DNA probes. A mixture of locus-specific probes for loci 22q11.21 (HIRA) and 22q13.33 (ARSA) of chromosome 22 manufactured by Cytocell Aquarius (UK) was used.

**Figure 2:**
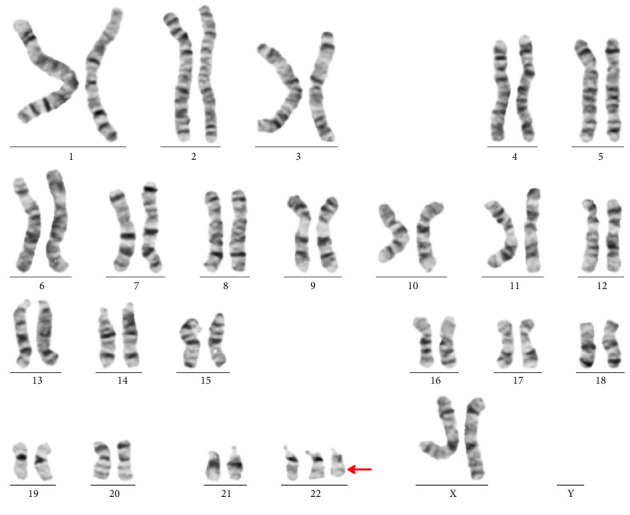
The patient's karyotype using G-band analysis shows a supernumerary derivative chromosome 22 (arrowhead).

**Figure 3:**
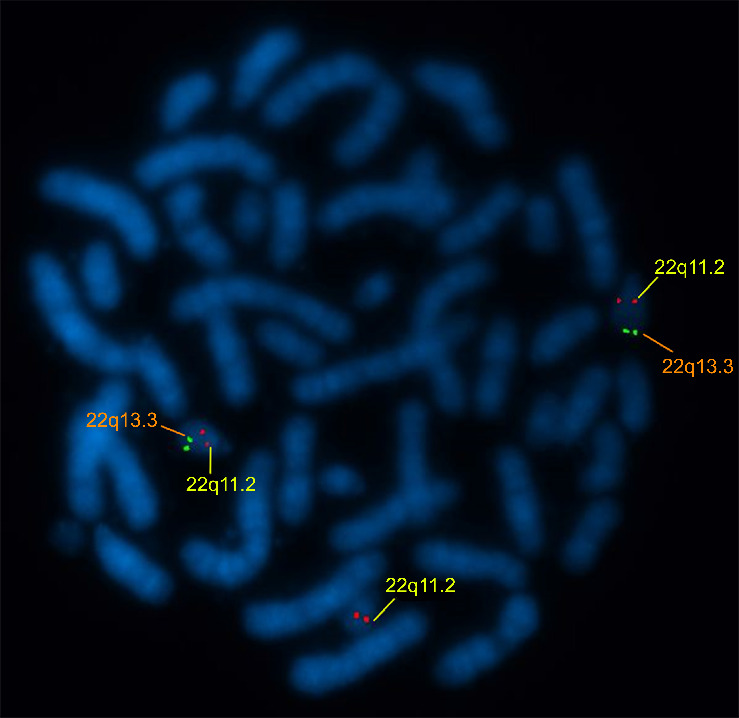
Fluorescent in situ hybridization image with der(22)t(11; 22); green signals show the 22q13.3 and red signals show the 22q11.2 region.

The patient's karyotyping shows a supernumerary chromosome 22, der(22)t(11; 22) (Figure 2). The absence of the telomeric region 22q13.3 on derivative chromosome 22 was clarified by FISH (ish 22(q11.2q13.3) (HIRA+,ARSA−) Figure 3.

**Figure 4:**
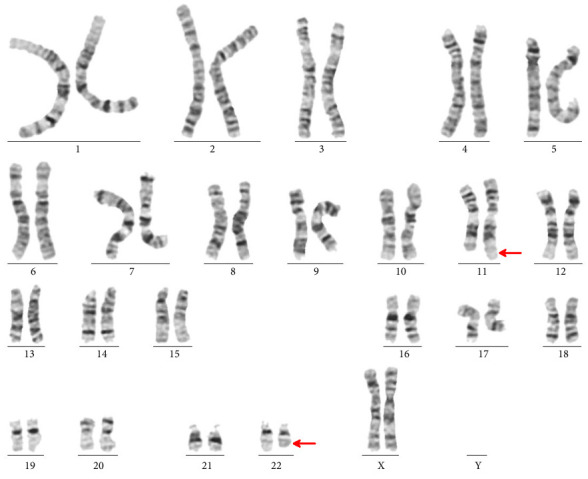
The mother's karyotype shows a balanced non-Robertsonian translocation between chromosome 11 and chromosome 22 (arrowheads).

To ascertain the origin, the supernumerary marker chromosome cytogenetic testing was performed on both parents. The father had a normal karyotype. and the mother was found to be a carrier of balanced reciprocal translocation 46, XX, t(11; 22)(q23.3; q11.2) (Figure 4).

## 3. Discussion

The presented case report underscores the challenges of prenatal ES diagnosis, particularly in the absence of family history or gross malformations.

The syndrome is known to be characterized by polymorphism of prenatal and postnatal signs of varying severity [3–23]. Even the most commonly observed heart, kidney defects, and diaphragmatic hernia are detected in utero in no more than 30% of cases [12]. Varying degrees of prenatal expressiveness were also noted for other malformations and facial dysmorphia [7, 11–23], which is partly explained by oligohydramnios [6, 20] and complicated timely ES detection. Although posterior fossa defects are a fairly common prenatal finding, such defects are detected in approximately half of cases [11–14, 17] and there are ES cases without any adverse perinatal event [5–10, 14, 21–23]. Prenatal diagnosis is problematic if the developmental defects are not amenable to echographic imaging.

Prenatal diagnosis is especially challenging in families without a known history of t(11; 22). The potential effectiveness of noninvasive prenatal testing (NIPT) for ES was shown, but mainly in cases with recognized carriership [14, 24] and NIPT, sensitivity for microduplication syndromes is still insufficient due to the small size of the involved chromosomal region and the presence of repetitive sequences. Preimplantation genetic testing may prevent affected pregnancies but only for known carriers and it is not applicable for population-wide screening.

Given that diagnostic testing remains the gold standard for the prenatal diagnosis of ES, routine prenatal screening is an important tool for identifying high-risk groups for prenatal karyotyping. In the case of ES, the generally accepted view is that there are neither sensitive markers nor specific prenatal echographic characteristics of ES. Despite the relatively small number of publications with the description of prenatal signs of the syndrome, according to our knowledge, 28 cases summarized in several reviews and case reports [11–21], they clearly demonstrate the variability of the ultrasound picture in the second trimester. Nevertheless, the informativeness of first-trimester screening, in particular for NT, has not been systematically analyzed.

Out of eight published cases with available first-trimester data [11–18], NT was reported as increased in five [11–13, 16, 18]. In the series of Walfisch et al. [11], among five described cases, NT was measured in one and found to be 4 mm (2.94 MoM). Similar increases were reported by Kee et al. [16] (NT 3.3 mm, with ductus venosus flow abnormality) and Xu et al. [18] (NT 3.3 mm). Piwowarczyk et al. [12] described two first-trimester cases, one with NT of 3.9 mm, while in the other, it was stated only that no soft markers were detected and the NT size was not specified. In the series of Hao et al. [13], which included six cases, NT measurements were not provided; however, the authors noted increased NT in one case and an increased nuchal fold in two others. In the case reported by Taddei et al. [15], NT was at the upper limit for 12 + 2 weeks (2.4 mm, 90th percentile; CRL 65.1 mm), leading to karyotyping on the basis of maternal age risk. In two additional reports, NT values were within the normal range: 2.0 mm and 1.7 mm, respectively [14, 17].

Biochemical maternal serum markers did not appear to be typical for the syndrome. In Walfisch et al. series of five ES cases [11], second-trimester biochemical screening results were available in four of them and markers were within normal limits. The first-trimester screening was available in one case, and both markers were less than < 0.5 MoM: PAPP-A-0.25; βhCG-0.49. Similar results were shown in another case report presented by Kilijanova et al. [17]: PAPP-A-0.28, βhCG-0.66. In a series of six ES cases described by Hao et al. [13], three had biochemical screening and in all results were unchanged. Available data fail to suggest a consistent specific pattern. Most likely altered biochemical markers in those two cases were associated with the presence of other abnormalities. Nasal bone aplasia also does not appear to be the definitive characteristic of the syndrome. This marker was noted in two of the six cases in the Hao et al. series [13]. Given that other descriptions of first-trimester ultrasound screening did not report this finding, it might be assumed that it was not observed.

In our case, among the first-trimester markers, only an increased NT was present—3.2 mm at 12 weeks. That is, together with our report, increased NT in ES fetuses was observed in 6 out of 9 cases (66.7%), excluding the case with a borderline value. Notably, NT thickening was the most prominent feature in fetuses with complete trisomy 22 [25]. Although the rarity of the ES and limited prenatal data in the first-trimester preclude reliable estimates of its diagnostic accuracy, these results are noteworthy and suggest that NT measurement may serve as a valuable tool for identifying at-risk fetuses, warranting further prospective studies. This conclusion is supported by a recent study that examined the outcomes associated with fetal NT between 3.0 and 3.4 mm in the first trimester and found that with NT values in this range, the rate of chromosomal abnormalities was 16.7%. The same study showed that in fetuses with NT from 3.0 to 3.4 mm, the frequency of adverse outcomes, although two times lower than with NT ≥ 3.5 mm, still amounted to 32.1% [26].

Many carriers of balanced t(11; 22) are identified only after reproductive challenges or the birth of an affected child as was the case in our report. The translocation t(11; 22) is not only the most common recurrent non-Robertsonian constitutional translocation in humans. Based on its geographic distribution [3], the frequency of this mutation may be much higher than ∼1 in 16,000 as was estimated in a single study [27]. In addition to the implications for prenatal diagnosis, there is another important aspect connected with timely detection of carriers since several studies suggest long-term health risks for carriers of balanced t(11; 22) translocations, including an increased risk of breast cancer and other malignancies [28–31]. This underscores the importance of genetic counseling for both reproductive and broader health management.

## 4. Conclusion

This case highlights that isolated NT thickening may represent the only detectable prenatal sign of ES. Although current evidence is insufficient to draw definitive conclusions given the limited first-trimester data in the literature, the recurrence of this finding across reports suggests that NT measurement could provide a valuable tool for identifying at-risk pregnancies. Evaluation in larger cohorts and future prospective studies will be essential to establish the sensitivity and specificity of this marker for early diagnosis of the syndrome and for the timely recognition of balanced translocation carriers.

## Data Availability

The data that support the findings of this study are available from the corresponding author upon reasonable request.

## Nomenclature

ES: Emanuel syndrome
NT: Nuchal translucency
FISH: Fluorescent *in situ* hybridization
MRI: Magnetic resonance imaging
NIPT: Noninvasive prenatal testing
